# Reviewed article: Lima LV, Pavinati G, Silva RGT, Queiroz IG, Oliveira GLA, Durães SMB, López MAA, Alves KBA, Magalhães FB, Magnabosco GT. Leprosy elimination phase in Alagoas, Brazil, 2001-2022: an ecological study. Epidemiol Serv Saude. 2025;34:e20240255. Doi: 10.1590/S2237-96222024v34e20240255.en

**DOI:** 10.1590/S2237-96222024v34e20240255.a

**Published:** 2025-04-11

**Authors:** Antônio Carlos Vieira Ramos

**Affiliations:** 1 Universidade do Estado de Minas Gerais, Fundação de Ensino Superior de Passos, Passos, MG, Brasil

## Peer review A

## Questionnaire

Does the manuscript contain new and significant information to justify publication? No

Does the Abstract (Summary) clearly and accurately describe the content of the article? Yes

Is the problem significant and concisely stated? No

Are the methods described comprehensively? No

Are the interpretations and conclusions justified by the results? No

Is adequate reference made to other work in the field? Yes

## Manuscript Structure

Length of article is: Adequate

Number of tables is: Adequate

Number of figures is: Adequate

Please state any conflict(s) of interest that you have in relation to the review of this paper (state “none” if this is not applicable).

None

Interest: Good

Quality: Good

Originality: Good

Overall: Good

Recommendation: Major Revisior

## Comments

O manuscrito intitulado “Estágio de eliminação da hanseníase nos municípios de Alagoas, Brasil, 2001-2022: estudo ecológico com emprego da Leprosy Elimination Monitoring Tool” apresenta como objetivo analisar o estágio de eliminação da hanseníase nos municípios de Alagoas, Brasil, no período de 2001 a 2022. Como comentários gerais, o manuscrito apresenta relevância científica, ao analisar o panorama epidemiológico da hanseníase nos municípios do estado de Alagoas, através da utilização da análise espacial e temporal, bem como, apresenta caráter inédito, por utilizar a Leprosy Elimination Monitoring Tool, ferramenta ainda pouco utilizada no cenário Brasieiro.

Apesar de sua relevância, alguns aspectos necessitam ser melhor elucidados. A introdução não contextualiza o objeto de investigação (Leprosy Elimination Monitoring Tool); algumas escolhas metodológicas precisam ser esclarecidas e a discussão precisa se ater mais aos resultados do estudo. Sobre esses aspectos, recomendo “Revisões Maiores” ao manuscrito. 

Abaixo, comentários menores que subsidiam a presente decisão. 

Comentários menores:

Introdução

- Na introdução do manuscrito, ao mencionar os progressos no processo de eliminação da hanseníase, os autores citam as estratégias globais de enfrentamento propostas pela Organização Mundial da Saúde (OMS), contudo, não mencionam nenhuma estratégia nacional de enfrentamento. Recomendo aos autores que, complementarmente a apresentação das estratégias globais, que tragam as estratégias nacionais, fazendo uma reflexão crítica de sua importância no controle da hanseníase no Brasil.
- Recomendo aos autores que façam uma apresentação da “Leprosy Elimination Monitoring Tool”. Apesar dos autores apresentarem a ferramenta nos Métodos, creio que sua descrição deva ser feita na introdução, mencionando aspectos relacionados a sua criação e aplicabilidade.
- Quais são as evidências da literatura sobre a aplicação dessa ferramenta? O que a literatura científica aponta sobre sua aplicação? E a nível nacional, essa ferramenta já foi utilizada? Recomendo aos autores que tragam uma breve revisão de literatura discorrendo sobre as evidências da aplicação da Leprosy Elimination Monitoring Tool a contexto internacional e nacional.

Métodos

- De acordo com a descrição do item “População, variáveis e métodos de limpeza”, os autores utilizaram dois bancos de dados:
- A base estadual de dados, com os casos de hanseníase notificados como “caso novo”, com diagnóstico de 2001 a 2022, com a construção de duas varáveis: número de casos novos de hanseníase na população ≥ 15 anos e número de casos novos de hanseníase na população < 15 anos;
- O painel de indicadores, considerando o recorte temporal de 2010 a 2022, utilizando os indicadores: taxa de prevalência dos casos em tratamento, taxa de detecção de casos novos na população geral, taxa de detecção de casos novos na população < 15 anos.
- Em “Métodos estatísticos”, é descrito que as análises de séries temporais e análises espaciais foram realizadas com o conjunto de dados de 2010 a 2022, ao passo que, com os dados de 2001 a 2022, realizouse a categorização dos municípios pela Leprosy Elimination Monitoring Tool.
- Meu questionamento é o seguinte: porque os autores não utilizaram um único conjunto de dados para a realização de todas as análises (temporais, espaciais e aplicação da Leprosy Elimination Monitoring Tool)?
- Por exemplo, com o conjunto de dados de 2001 a 2022 (maior série histórica), além da construção das duas variáveis (número de casos novos de hanseníase na população ≥ 15 anos e número de casos novos de hanseníase na população < 15 anos) os autores poderiam calcular todos os indicadores do painel de indicadores (taxa de prevalência dos casos em tratamento, taxa de detecção de casos novos na população geral, taxa de detecção de casos novos na população < 15 anos), utilizando a base estadual de dados e a população do Instituto Brasileiro de Geografia e Estatística (IBGE) para a construção dos indicadores, de modo que, com um único banco de dados poderiam realizar todas as análises, com uma série temporal maior (2001 a 2022).
- Gostaria que os autores justificassem melhor sua escolha por dois bancos de dados.
- Resultados
- No último parágrafo dos Resultados, os autores informam que: “A categorização dos municípios pelos estágios de eliminação foi apresentada nos mapas referentes a 2006 (Figura 3A), 2011 (Figura 3B), 2016 (Figura 3C) e 2022 (Figura 3D)”. Sendo o período considerado de 2001 a 2022, qual foi o critério da categorização apresentada (2006, 2011, 2016 e 2022). Recomendo justificar no manuscrito.

Discussão

- No segundo parágrafo da discussão, os autores referem que: “Embora a tendência de declínio dos indicadores da hanseníase esteja consoante a outros estudos nacionais(13-15, 18), a doença apresenta altas taxas de detecção em regiões de pobreza e desigualdade social”. Quais são as regiões de pobreza e desigualdade social de Alagoas que apresentam altas taxas de detecção? Recomendo os autores descreverem com mais detalhes esse resultado, sobretudo os municípios para justificar essa afirmação.
- De maneira geral, os autores trazem importantes reflexões sobre aspectos de eliminação da doença e o papel dos determinantes sociais, entretanto, pouco articulam essas reflexões com seus resultados. Os resultados das figuras 2 e 3 foram pouco discutidos. Especialmente os resultados da figura 2, foram apresentados municípios hiperendêmicos, assim como, aglomerados Alto-Alto, fatores esses não discutidos.
- A ausência de casos em municípios não pode levantar a hipótese de subnotificação? Esse dado poderia ser melhor discutido.
- Qual a contribuição do estudo para o controle da hanseníase a nível local e nacional?

Em linhas gerais, recomendo que os autores discutam com maior aprofundamento seus resultados.

